# Pediatric population (aged 3-11 years) received primary inactivated SARS-CoV-2 vaccination prior to infection exhibiting robust humoral immune response following infected with Omicron variant: a study conducted in Beijing

**DOI:** 10.3389/fimmu.2023.1269665

**Published:** 2023-09-27

**Authors:** Jing Li, Jingjing Li, Shuzhi Dai, Li Dang, Lin Wang, Ling Cao, Xiaobo Chen, Ying Wang, Menglei Ge, Weijie Liu, Qinwei Song, Wenjian Xu, Lijuan Ma

**Affiliations:** ^1^ Department of Clinical Laboratory, Children’s Hospital, Capital Institute of Pediatrics, Beijing, China; ^2^ Department of Outpatient Treatment Center, Children’s Hospital, Capital Institute of Pediatrics, Beijing, China; ^3^ Department of Child Health Care, Children’s Hospital, Capital Institute of Pediatrics, Beijing, China; ^4^ Department of Respiratory, Children’s Hospital, Capital Institute of Pediatrics, Beijing, China; ^5^ Department of Endocrinology, Children’s Hospital, Capital Institute of Pediatrics, Beijing, China

**Keywords:** vaccination, COVID-19, children, SARS-CoV-2 infection, neutralizing antibody

## Abstract

**Objective:**

Analysis of SARS-CoV-2 IgG antibody and neutralizing antibody levels following SARS-CoV-2 infection in children aged 3-11 years, comparing those who had received the inactivated SARS-CoV-2 vaccine to those who were unvaccinated prior to infection, provides evidence for public health centers in formulating vaccination strategies and control policies.

**Methods:**

A study was conducted on children who visited the Children’s Hospital, Capital Institute of Pediatrics from January 10, 2023 to March 31, 2023 (Beijing, China). Participants or their guardians completed a survey questionnaire providing information about their SARS-CoV-2 infection history and vaccination status. Serum samples were collected for testing of SARS-CoV-2 immunoglobulin G (IgG) and neutralizing antibodies (Nabs), which were performed using chemiluminescence immunoassay.

**Results:**

The study included 1,504 children aged 3-11 years with previous SARS-CoV-2 infection history. Among the 333 unvaccinated children, the serum SARS-CoV-2 IgG antibody level was median 2.30 (IQR, 1.27-3.99). However, children received one dose (78 cases) and two doses (1093 cases) of the inactivated vaccine prior to infection showed significantly higher SARS-CoV-2 IgG antibody levels, with values of median 10.11 (IQR, 8.66-10.93) and median 10.58 (IQR, 9.79-11.07), respectively. As to the unvaccinated children, 70.3% (234/333) were negative for SARS-CoV-2 Nabs, which were less than 6.00AU/ml. The remaining 29.7% (99/333) showed relatively low levels of Nabs, ranging from 6.00 to 50.00AU/ml. In contrast, for children who had received two doses of vaccine prior to infection, an overwhelming 99.3% (1086/1093) exhibited high levels of Nas in the range of 100.00-120.00 AU/ml. Remarkably, these elevated Nab levels persisted for at least a period of 3 months post-infection in children who had received two doses of inactivated SARS-CoV-2 vaccine prior to infection, regardless of age or sex and vaccine manufacturer.

**Conclusion:**

The administration of two doses of inactivated SARS-CoV-2 vaccine prior to infection has been shown to significantly enhance humoral immunity following SARS-CoV-2 infection in pediatric populations, producing adequate Nabs that persist at elevated levels for up to 3 months post-infection. For unvaccinated children who displayed weak humoral immunity following a primary natural infection, timely vaccination is recommended to bolster their immunization protection. The findings underscore the importance of vaccination in strengthening immune responses and protecting pediatric populations against SARS-CoV-2 infection.

## 1 Introduction

Since December 2019, the world has been grappling with the emergence and relentless spread of coronavirus disease 2019 (COVID-19), a highly contagious respiratory illness caused by the novel severe acute respiratory syndrome coronavirus 2 (SARS-CoV-2). This unprecedented pandemic has presented significant challenges to global health systems and economies (1, 2). In response to the dynamic nature of the virus, the World Health Organization (WHO) has been monitoring and categorizing different variants of the coronavirus. The repertoire of identified variants encompasses a range of strains, notably the Alpha, Beta, Gamma, and Delta variants, and most recently, the Omicron variant. The Omicron variant, scientifically known as B.1.529, was first identified in South Africa in early November 2021 (3). Since then, it has been a subject of intense scrutiny due to its high number of mutations and potential implications for transmissibility, immune escape, and vaccine effectiveness (4).

The Chinese government has applied a series of epidemic prevention and control measures, including SARS-CoV-2 vaccination, routine SARS-CoV-2 nucleic acid testing, wearing masks in public places, and quarantine of confirmed cases. The strategic implementation of vaccination has emerged as a critical weapon in empowering governments across the globe to effectively combat and control the transmission of SARS-CoV-2. The immunogenicity of two inactivated vaccines, namely CoronaVac (Sinovac, Beijing, China) and BBIBP-CorV (Beijing Institute of Biological Products, Beijing, China), were undertaken through a meticulous phase 1/2 trial that specifically targeted individuals aged 3–11 years. The comprehensive assessment of these inactivated vaccines revealed a remarkable outcome, as they unequivocally demonstrated their ability to provoke strong immune responses within the vaccinated cohort. A seroconversion ratio of 100% was observed across all vaccinated individuals, precisely 28 days after the administration of the second vaccine dose (5, 6). This extraordinary efficacy in eliciting humoral responses holds paramount significance in conferring protective immunity, thus establishing a solid foundation to effectively safeguard and fortify the younger age group against the perils of SARS-CoV-2. Subsequently, the vaccines were approved for use in children older than 3 years of age in China from November 2021, with primary immunization consisting of two doses administered over a 28-day period. A recent study conducted in Chile demonstrated that a complete primary immunization for CoronaVac in children aged 6–16 years provided effective protection against hospitalization and severe COVID-19 (7).

The SARS-CoV-2 Omicron variant has been circulating extensively in China since December 2022, with BA.5.2 and BF.7 being the prevalent circulating strains (8). More importantly, children were susceptible to the virus. Real-world evaluations investigating the humoral immune response triggered by Omicron variant infection in pediatric populations, who have received the inactivated SARS-CoV-2 vaccine, are exceptionally scarce. In the quest for comprehensive pandemic management and control, the monitoring of serological antibodies in children emerges as a pivotal aspect. With the aim of shedding light on this critical subject, an investigation was undertaken in this study, concentrating on the analysis of serum SARS-CoV-2 antibody levels in a distinct population of children aged 3-11 years. Within the scope of the study, a comparison was conducted to evaluate the SARS-CoV-2 IgG antibody and Nab levels within 3 months post-SARS-CoV-2 infection. The distinction laid in segregating the participants into two cohorts: those who had received the inactivated SARS-CoV-2 vaccine and those who had not undergone vaccination before falling prey to the virus. The study aimed to furnish public health agencies, guiding vaccination strategies and formulating control policies.

## 2 Materials and methods

### 2.1 Survey questionnaire of previous SARS-CoV-2 infection

​ The survey questionnaire embarked on a comprehensive exploration, including the SARS-CoV-2 previous infection history and vaccination status. Assertion of SARS-CoV-2 infection required confirmation of prior positive SARS-CoV-2 nucleic acid or antigen tests. Meanwhile, the SARS-CoV-2 vaccination status survey included the number of doses received, along with the vaccine manufacturer. The interval between the administration of the last vaccine dose and the subsequent occurrence of SARS-CoV-2 infection was investigated. The survey questionnaire offered alternative options for all its probing inquiries, ensuring comprehensive coverage. However, for aspects not mentioned in the alternative items, the questionnaire also provided an open avenue for participants to fill in relevant details. To ensure accuracy and fidelity to the data, the questionnaire was diligently self-reported. For 8-11 years, the mantle of self-reporting rested on their understanding, while guardians took on the responsibility of representation for children aged 3-7 years. Children who answered the questionnaire visited the outpatient department of Children’s Hospital, Capital Institute of Pediatrics between January 10, 2023 and March 31, 2023 and their venous blood samples were drawn for clinical items examination. Remaining serum specimens from eligible children were further tested for SARS-CoV-2 IgG and Nabs. In the study, all the participants’ data were anonymously reported and the protocol was approved by the Ethics Committee of Capital Institute of Pediatrics (approval number SHERLL2023016).

### 2.2 Blood sample collection

Venipuncture was undertaken to collect approximately 3 ml of peripheral blood from each participant using coagulation tubes. The blood samples underwent centrifugation at 3,000 rpm/min for a duration of 10 min, effectively separating the serum from the cellular components. It was stored at -80 °C for further analyses.

### 2.3 SARS-CoV-2 IgG and Nabs detection

SARS-CoV-2 IgG and Nabs were analyzed using the reagent matching the Maccura i1000 automatic chemiluminescence analyzer (Maccura Biotechnology Co., Ltd., Chengdu, China). Detection of IgG antibodies reactive to the recombinant RBD antigen of the SARS-CoV-2 Spike protein. It was semiquantitative and the concentration was recorded as S/CO (RLU of samples to be tested/cutoff), with results of ≥1.000 S/CO represented as positive. Detection of Nabs that may block the interaction between the RBD domains of SARS-CoV-2 and human ACE2. Briefly, the specimen was incubated with a pre-coated magnetic particle, which allowed the Nab to bind the RBD domain of SARS-CoV-2 on the magnetic particles. Then, the hACE2 labelled with acridinium ester was added, it will bind to the pre-coated magnetic particles at the RBD binding sites that remains unblocked by the neutralizing antibody. The Nabs assay is quantitative and the concentration was recorded as AU/ml, with the level ≥6.000 represented a positive result. The quantitative detection range was 3.00 to 120.00 AU/ml.

## 3 Statistical analysis

Continuous variables were presented as median and interquartile range (IQR). SARS-CoV-2 IgG and Nab levels were compared using the Mann‐Whitney U test or Wilcoxon test. Categorical variables were expressed as number and percentage. Nabs grades in different vaccination status post-infection were compared using the Chi-squared test. P < 0.05 was considered statistically significant. The statistical analysis and illustration of graphs were performed by GraphPad Prism 8.0 software (GraphPad Software Inc., San Diego, CA, USA).

## 4 Results

### 4.1 Participants’ characteristics

**Figure 1:**
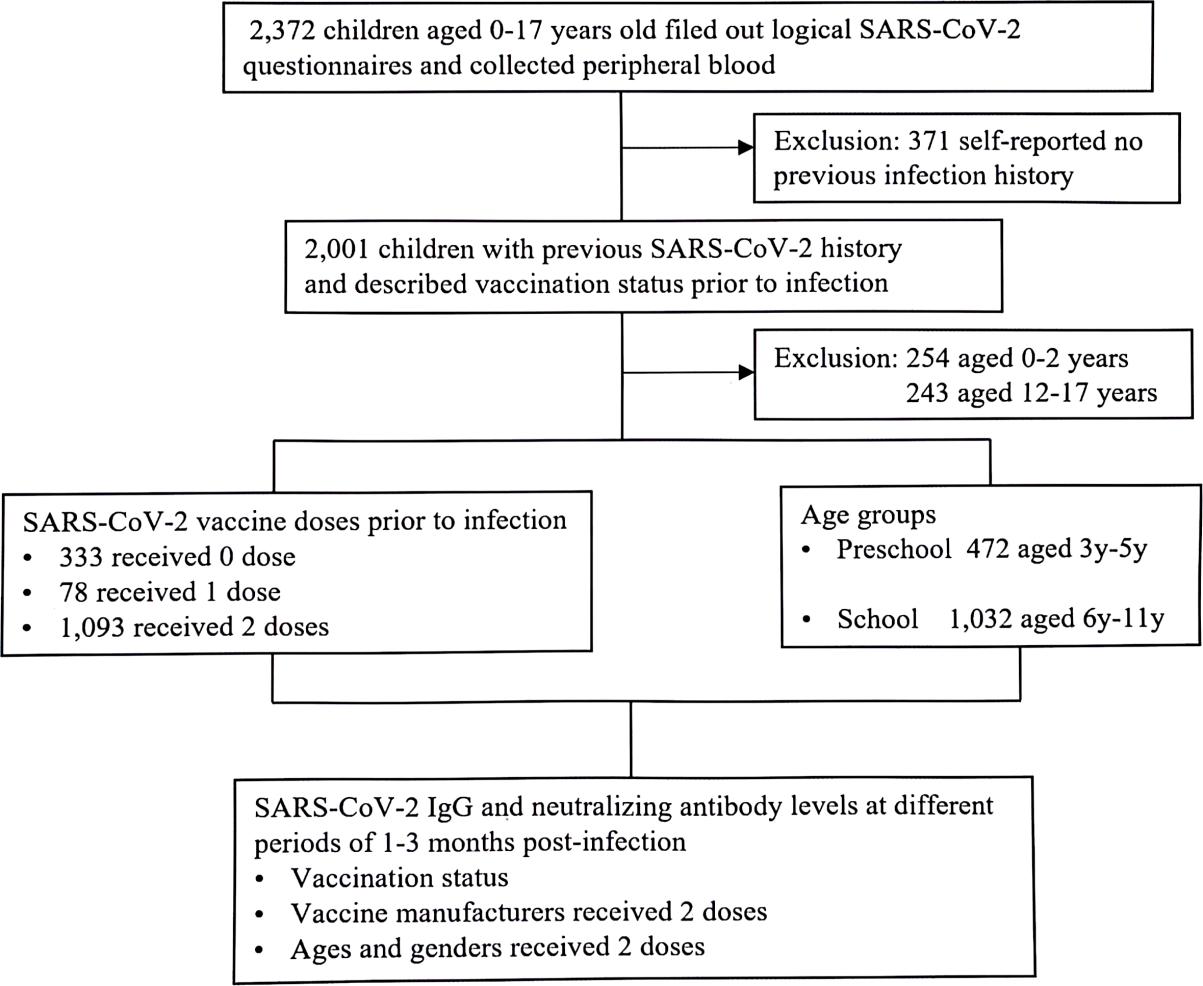
Study involvement (January 2023-March 2023). Children visited to the outpatient of Children’s Hospital, Capital Institute of Pediatrics, with previous SARS-CoV-2 infection. All the participants tested for SARS-CoV-2 serological assay.

The study enrolled a sizeable cohort of 1,504 children, aged 3 to 11 years, each with a confirmed history of previous SARS-CoV-2 infection, including 723 boys and 781 girls. To ensure the accuracy of the study, children with autoimmune or hematological diseases were excluded. Based on the age of the participants, they were divided into a 3-5 years preschool group and a 6-11 years school age group. With a keen concentration on the levels of SARS-CoV-2 IgG and Nabs between SARS-CoV-2 vaccine doses and subsequent infection, the study attempted to analyze the impact of prior vaccination status on the immune responses of young participants (**Figure 1**; **Table 1**). Furthermore, SARS-CoV-2 antibody expression was analyzed at different periods of 1-3 months post infection in two doses vaccinated individuals prior to infection of different vaccine manufacturers, ages and genders of children (**Table 2**).

**Table 1:** Characteristics and vaccination status of SARS-CoV-2 infected children (N%).

Characteristic	Total	3y-5y	6y-11y
	n=1,504	n=472	n=1,032
Sex			
Male	723 (48.1)	197 (41.7)	526 (50.9)
Female	781 (51.9)	275 (58.3)	506 (49.1)
Vaccination dose before infection			
0 dose	333 (22.1)	218 (46.2)	115 (11.1)
1 dose	78 (5.2)	46 (9.7)	32 (3.1)
2 doses	1,093 (72.7)	208 (44.1)	885 (85.8)
Vaccine manufacturer			
Non-vaccination	333 (22.1)	218 (46.2)	115 (11.1)
CoronaVac	771 (51.3)	177 (37.5)	594 (57.6)
BBIBP-CorV	400 (26.6)	77 (16.3)	323 (31.3)
Months interval between the second dose and the infection			
0 month	333 (22.1)	218 (46.2)	115 (11.1)
3-6 months	78 (5.2)	46 (9.7)	32 (3.1)
>6 months	1,093 (72.7)	208 (44.1)	885 (85.8)

Data were presented as number (percentage). Based on vaccination status prior to infection, participants were divided into nonvaccinated, one dose and two doses vaccinated groups. According to the age, recipients were divided into 3-5 years old preschool aged and 6-11 years old school aged groups.

**Table 2:** Distribution periods of blood collection post SARS-CoV-2 infection in 2 doses vaccinated children (N%).

Characteristic	Total	1 month	2months	3 months
	n=1,093	n=422	n=448	n=223
Sex				
Male	561 (51.3)	225 (53.3)	227 (50.7)	109 (48.9)
Female	532 (48.7)	197(46.7)	221 (49.3)	114 (51.1)
Ages				
3-5y	208 (19.0)	94 (22.3)	84 (18.8)	30 (13.5)
6-11y	885 (81.0)	328 (77.7)	364 (81.2)	193 (86.5)
Vaccine manufacturer				
CoronaVac	720 (65.9)	334 (79.1)	269 (60.0)	117 (52.5)
BBIBP-CorV	373 (34.1)	88 (20.9)	179 (40.0)	106 (47.5)

Data were presented as number (percentage). According to the intervals between blood collection and onset of symptoms of SARS-CoV-2 infection, it was divided into 3 periods from 1-3 months post infection.

The history of prior SARS-CoV-2 infections among 1,504 children predominantly unfolded during December 2022. Notably, the infection rates exhibited variations across the early, middle, and late phases of that month, standing at 29.7% (446/1504), 45.0% (677/1504), and 18.9% (284/1504), respectively. Moreover, an additional 6.4% (97/1504) of infections occurred in the early days of January 2023. Within this cohort, 1,171 children received the inactivated SARS-CoV-2 vaccine prior to infection. Participants vaccinated with Corona Vac and BBIBP-CorV were 65.8% (771/1171) and 34.2% (400/1171), respectively.

### 4.2 SARS-CoV-2 IgG and Nabs levels in children within 3 months post-infection

**Figure 2:**
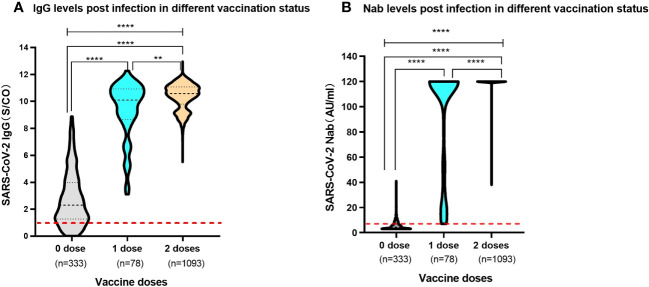
Levels of SARS-CoV-2 IgG antibody and Nab in participants post infection with different previous vaccination status **(A)**. SARS-CoV-2 IgG antibody levels. The red dashed line shows a threshold value of 1.00 S/CO. **(B)**. SARS-CoV-2 Nab levels. The red dashed line shows a threshold value of 6.00 AU/ml. Above data were presented as median values along with interquartile ranges in the violin plot, and analyzed using the Mann‐Whitney U test. The symbols meaning, ** P<0.01, **** P<0.0001.

Of the 1,504 children, 333 recipients were unvaccinated prior to SARS-CoV-2 infection. Of the vaccinated individuals, 78 and 1,093 children received one and two doses of SARS-CoV-2 vaccine, respectively. The serum SARS-CoV-2 IgG antibody level of unvaccinated children was median 2.30 (IQR, 1.27-3.99). Conversely, children who received one dose and two doses vaccine prior to infection had a SARS-CoV-2 IgG antibody level of median 10.11 (IQR, 8.67-10.93) and median 10.58 (IQR, 9.97-11.07), respectively. The levels of SARS-CoV-2 IgG antibody in vaccinated children surpassed those observed in their unvaccinated counterparts with a remarkable level of statistical significance (P<0.0001). Moreover, children who had previously received two doses of vaccine showed higher levels of SARS-CoV-2 IgG antibodies following infection than those who had received one dose (P=0.005). For the study into the realm of Nabs, the SARS-CoV-2 Nabs in unvaccinated children was median 4.34AU/ml (IQR 3.00-6.89). Whereas children vaccinated with one dose and two doses had an elevated Nabs, which reached at median 120.00AU/ml (IQR 56.47-120.00) and median 120.00AU/ml (IQR 120.00-120.00), respectively. The levels of SARS-CoV-2 Nab exhibited a significant difference among the participants associated with previous vaccination status (P<0.0001). Pre-vaccinated children showed substantially elevated levels of SARS-CoV-2 Nabs after being infected with SARS-CoV-2 compared to children who had not received prior vaccination (**Figures 2A, B**).

Further examination was conducted to delve into the relationship between the number of SARS-CoV-2 vaccine doses and the expression levels of SARS-CoV-2 Nabs post-infection. For the unvaccinated children prior to SARS-CoV-2 infection, a substantial majority, accounting for 70.3% (234/333) of the cohort, exhibited negative Nabs, registering levels below 6.00 AU/ml. Additionally, the remaining 29.7% (99/333) demonstrated comparatively low levels, ranging from 6.00 to 50.00 AU/ml. Children who had received one dose of the SARS-CoV-2 inactivated vaccine prior to infection exhibited diverse Nab levels. Specifically, 20.6% (16/78) displayed levels between 6.00 and 50.00 AU/ml, while a smaller fraction, representing 7.6% (6/78), fell within the 50.00-100.00 AU/ml range. Notably, the majority of this group, comprising 71.8% (56/78), boasted an immensely elevated level of Nabs, peaking at 100.00-120.00 AU/ml. The most remarkable revelation emerged in the group of children who had received two doses of the SARS-CoV-2 vaccines prior to infection. An overwhelming 99.4% (1086/1093) of these individuals demonstrated a robust humoral response, yielding high-level Nabs that clustered between 100.00 and 120.00 AU/ml. Significantly, the levels of neutralizing antibodies exhibited profound discrepancies among the different vaccination status groups(χ2 = 1401.505, P<0.0001) (**Table 3**).

**Table 3:** The relationship between Nab levels post SRAS-CoV-2 infection and vaccination status.

Vaccination status prior to infection	Number	SARS-CoV-2 Nabs(AU/ml , N/%)			
		<6.00	6.00 -<50.00	50.00-<100.00	100.00-120.00
0 dose	333	234 (70.3)	99 (29.7)	0 (0)	0 (0)
1 dose	78	0 (0)	16 (20.6)	6 (7.6)	56 (71.8)
2 doses	1093	0 (0)	1 (0.1)	6 (0.5)	1086 (99.4)

Data were presented as number (percentage). The relationship between the level of Nabs post SARS-CoV-2 infection and the vaccine status prior infection. According to the value of Nabs, it was divided into four subgroups from low to high levels.

### 4.3 SARS-CoV-2 antibody expression at different periods of 1-3 months post infection

**Figure 3:**
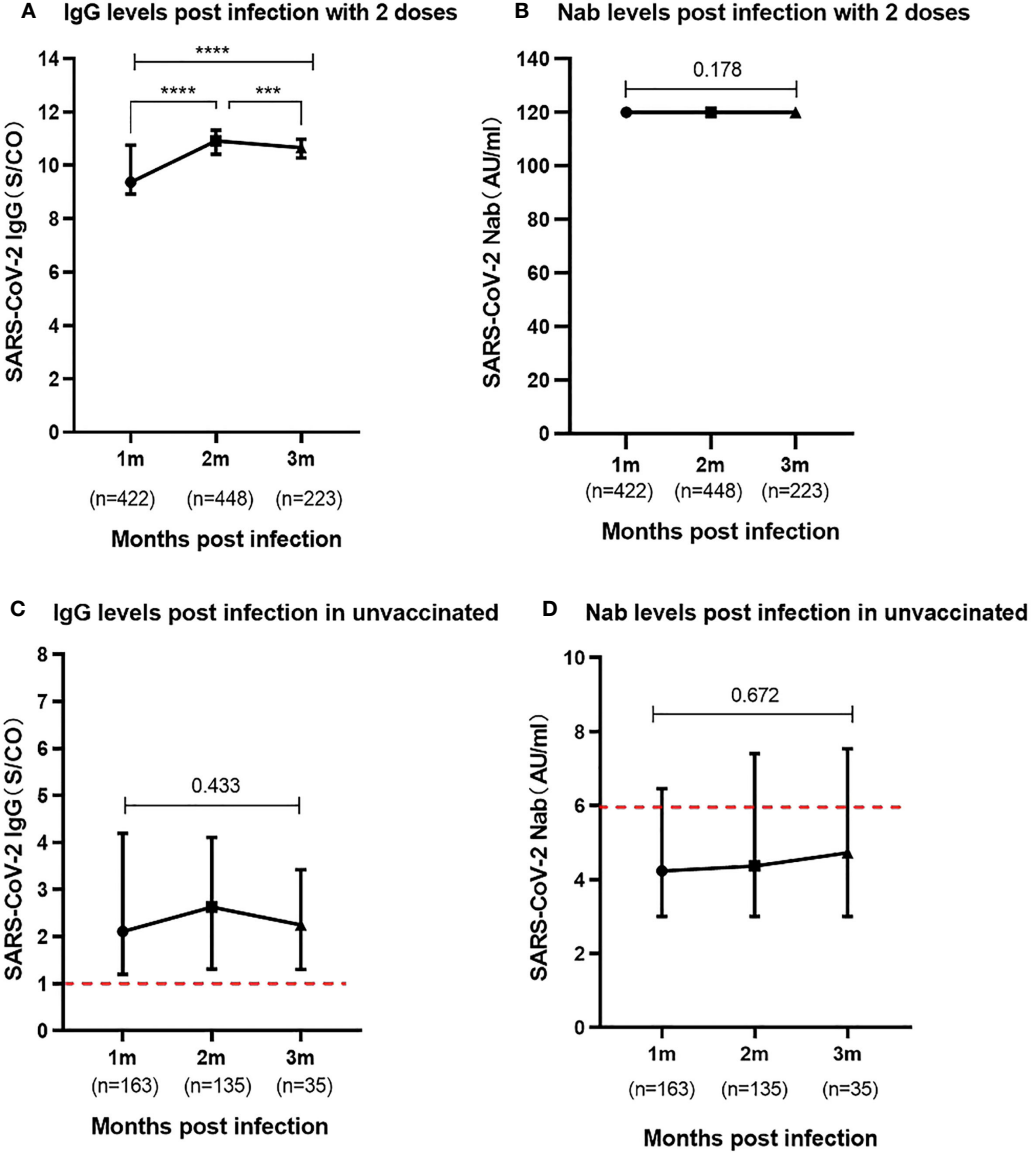
Levels of SARS-CoV-2 IgG and Nab during the 1-3 months post infection **(A)**. SARS-CoV-2 IgG antibody levels in participants previously received two vaccine doses. **(B)**. SARS-CoV-2 Nab levels in participants previously received two vaccine doses. **(C)**. SARS-CoV-2 IgG antibody levels in participants unvaccinated prior to infection. **(D)**. SARS-CoV-2 Nab levels in participants unvaccinated prior to infection. Above data were presented as median values along with interquartile ranges in the violin plot, and analyzed using the Mann‐Whitney U test. The symbols meaning, *** P<0.001, **** P<0.0001.

In the study, a substantial portion of the participants, comprising 72.6% (1,093/1,504), had completed the two-dose schedule of the inactivated SARS-CoV-2 vaccine prior to infection, with the time interval between the second dose and infection spanning over 6 months. To analyze the tendency of the immune response post-infection in those who received two doses vaccine and unvaccinated prior infection, participants were categorized into three distinct periods of 1-3 months post-infection. The 1-month, 2-month and 3-month periods indicated the 25-35 days, 55-65 days and 85-95 days intervals between blood collection and onset of symptoms of SARS-CoV-2 infection, respectively. For individuals who had undergone the two-dose vaccine schedule, the SARS-CoV-2 IgG antibodies revealed levels of median 9.36 (IQR, 8.90-10.75), median 10.91 (IQR, 10.41-11.31), and median 10.65 (IQR, 10.28-10.97) at 1-3 months post-infection, respectively. Notably, the IgG antibody levels displayed a remarkable trend, with levels higher in the last two months compared to the first month (P<0.0001). Whereas the levels of SARS-CoV-2 Nabs remained exceptionally elevated, consistently recording 120.00 AU/ml for the entire duration of 3 months post-infection in the two doses vaccinated individuals. Remarkably, no statistically significant difference was discerned in the serum levels of Nabs across different months of convalescence (P=0.178) (**Figures 3A, B**).

In children who were unvaccinated prior to infection, the study observed relatively low levels of SARS-CoV-2 IgG antibodies in the serum during the 1 to 3 months following infection. The results showed levels of median 2.11 (IQR, 1.20-4.19), median 2.63 (IQR, 1.31-4.11), median 2.25 (IQR, 1.30-3.42), respectively. Notably, the serum Nab levels in the unvaccinated group were particularly meager, with median Nabs falling below the positivity threshold at a median of 4.23-4.73AU/ml during the three-month post-infection period. There were no statistically significant differences in the levels of both SARS-CoV-2 IgG antibodies (P=0.433) and Nabs (P=0.672) at 1 to 3 months among these unvaccinated individuals (**Figures 3C, D**).

### 4.4 SARS-CoV-2 antibody levels in two doses vaccine of different manufactures

**Figure 4:**
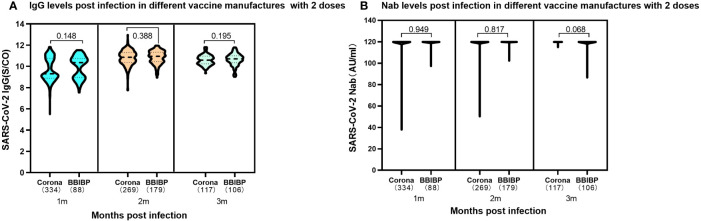
Levels of SARS-CoV-2 IgG antibody and Nab at 1-3 months post infection in participants previously received two doses either CoronaVac or BBIBP-CorV. **(A)**. SARS-CoV-2 IgG antibody levels. Corona Vac: 1 month, median 9.30 (IQR, 8.87-10.76); 2 months, median 10.85 (IQR, 10.37-11.29); 3 months, median10.59 (IQR, 10.24-10.96); BBIBP-CorV: 1 month, median 10.35 (IQR, 8.97-10.74); 2 months, median 10.94 (IQR, 10.44-11.34); 3 months, median 10.70 (IQR, 10.34-11.00); The SARS-CoV-2 IgG results showed no significant difference in the two vaccines at the same time periods with the P values of 0.148, 0.388 and 0.195 at 1, 2, 3 months following infection, respectively. **(B)**. SARS-CoV-2 Nab levels. Both CoronaVac and BBIBP-CorV vaccines, 1-3 months: median 120.00AU/ml (IQR, 120.00-120.00). The SARS-CoV-2 Nabs showed no significant difference in the two vaccines at the same time periods. The P values were 0.949, 0.817 and 0.068 at 1, 2, 3 months following infection, respectively. Above data were presented as median values along with interquartile ranges in the violin plot, and analyzed using the Wilcoxon test.

The study compared the humoral immune response of 1,093 children infected with SARS-CoV-2 who received two doses of different inactivated vaccines prior to infection. The expression of SARS-CoV-2 IgG antibodies was maintained at elevated levels in both Corona Vac and BBIBP-CorV vaccinated individuals, and they were similar at different periods of 1-3 months following SARS-CoV-2 infection. There was no significant difference in IgG antibody levels between the SARS-CoV-2 inactivated vaccines produced by the two different manufacturers at the same period (**Figure 4A**). Further analysis of the immune response on Nab levels showed that children who either received 2 doses of Corona Vac or BBIBP-CorV prior to infection could produce elevated levels of Nab peaking at median 120.00AU/ml (IQR, 120.00-120.00) during the 1 to 3 months following infection (**Figure 4B**).

### 4.5 SARS-CoV-2 antibody levels in two doses vaccine of different ages and genders

**Figure 5:**
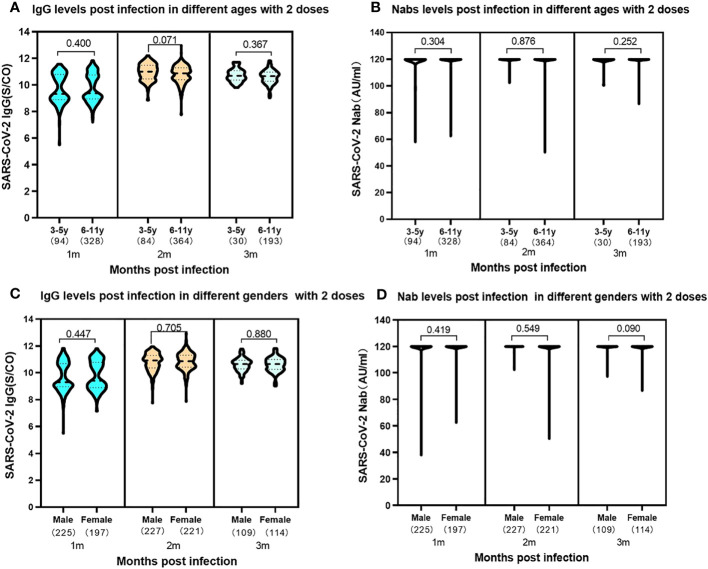
Levels of SARS-CoV-2 IgG antibody and Nab at 1-3 months post infection in participants of 3-5 years and 6-11 years previously received two vaccine doses. **(A)**. SARS-CoV-2 IgG antibody levels. 3-5 years: median 1month, 9.29 (IQR, 8.86-10.79); 2 months, median 11.00 (IQR, 10.45-1.47); 3 months, median 10.68 (IQR, 10.37-11.07); 6-11 years: 1 month, median 9.38 (IQR, 8.94-10.74); 2 months, median 10.87 (IQR, 10.39-11.29); 3 months, median 10.65 (IQR, 10.27-10.96). The SARS-CoV-2 IgG levels showed no significant difference in the two ages groups at the same time periods with the P values of 0.400, 0.071 and 0.367 at 1, 2, 3 months following infection, respectively. **(B)**. SARS-CoV-2 Nab levels. 1-3 months in the two ages: median 120.00AU/ml (IQR, 120.00-120.00). The SARS-CoV-2 Nabs showed no significant difference in the two ages groups at the same time periods. The P values were 0.304, 0.876 and 0.252 at 1, 2, 3 months following infection, respectively. Levels of SARS-CoV-2 IgG antibody and Nab at 1-3 months post infection in participants of different genders previously received two vaccine doses. **(C)**. SARS-CoV-2 IgG antibody levels. Male: 1month, median 9.29 (IQR, 8.95-10.70); 2 months, median 10.93 (IQR, 10.36-11.30); 3 months, median 10.68 (IQR, 10.37-11.07). Female: 1 month, median 9.38 (IQR, 8.94-10.74); 2 months, median 10.87 (IQR, 10.39-11.29); 3 months, median 10.65 (IQR, 10.30-10.93). The SARS-CoV-2 IgG levels showed no significant difference in the different genders at the same time periods. The P values were 0.447, 0.705 and 0.880 at 1, 2, 3 months following infection, respectively. **(D)**. SARS-CoV-2 Nab levels. Both male and female: median 120.00AU/ml (IQR, 120.00-120.00). The results showed no significant difference in different genders at the same time periods. The P values were 0.419, 0.549 and 0.090 at 1, 2, 3 months following infection, respectively. Above data were presented as median values along with interquartile ranges in the violin plot, and analyzed using the Wilcoxon test.

In the study, an exploration of the relationships between the SARS-CoV-2 antibody levels with gender and age, within the children received a complete two-dose vaccine regimen was conducted. Intriguingly, the results revealed no discernible significant difference in the expression levels of SARS-CoV-2 IgG and Nabs between boys and girls at 1-3 months following SARS-CoV-2 infection (**Figures 5A, B**). Moreover, the analysis expanded to encompass two distinct age groups within the vaccinated cohort. The data unveiled no disparity in the expression of SARS-CoV-2 IgG antibodies and Nabs between preschool children aged 3-5 years and school-aged children aged 6-11 years, who had completed two-dose SARS-CoV-2 vaccine regimen prior to infection (**Figures 5C, D**).

## 5 Discussion

The SARS-CoV-2 Omicron variant has been circulating extensively in China mainland since December 2022, 70% of cases in Beijing area were caused by the BF.7 variant (8). Children at different ages were mostly susceptible to the Omicron variant (9).

In our previous study, we conducted an investigation into the trends of SARS-CoV-2 IgG antibodies in children aged 3-11 years who had undergone primary immunization and had no previous history of SARS-CoV-2 infection. The study revealed that after receiving the second dose of the vaccine (10), SARS-CoV-2 IgG antibodies peaked at 1 month and remained at elevated levels for 3 months. Subsequently, the antibody levels gradually declined and closed to baseline after 6-7 months. Other research has also shown that vaccination with two doses of mRNA vaccine more than 6 months earlier, with no prior infection, offered limited protection against symptomatic Omicron infection, while demonstrated strong effectiveness in preventing severe conditions (11). In the current study, all children who received two doses of the inactivated SARS-CoV-2 vaccine had an interval of more than 6 months between the second dose and the infection. Consequently, the vaccine-induced antibodies against SARS-CoV-2 had nearly disappeared by the time of infection. Surprisingly, within 3 months post Omicron infection, the Nabs positive rate reached 100% in vaccinated individuals, and an impressive 99.4% of children who had received two doses prior to infection displayed elevated levels of Nabs, ranging from 100.00 AU/ml to 120.00 AU/ml. These findings highlight that children who completed two doses of the SARS-CoV-2 vaccine were capable of activating a robust humoral immune response, generating high levels of Nab, thereby offering effective protection against SARS-CoV-2 infection. Moreover, these high levels of Nab persisted in two doses vaccinated children for at least three months following infection.

We compared the post-infection humoral immune response to previous primary administration of either Corona Vac or BBIBP-CorV. The results showed that children who had previously received two doses of inactivated vaccine from different manufacturers were able to produce elevated levels of SARS-CoV-2 antibodies for at least three months after infection. In addition, we observed that children of different genders and ages who had received the primary SARS-CoV-2 vaccine prior to infection were capable of producing high levels of SARS-CoV-2 Nabs. Given that the majority of Chinese children over the age of 3 have received two doses of the inactivated SARS-CoV-2 vaccine prior to infection, the significant post-infection elevation in Nabs within the pre-vaccinated pediatric population played a crucial role in controlling the spread of the virus. Recent research has demonstrated that Omicron BA.1 infection after receiving three doses of the CoronaVac predominantly stimulates humoral immune memory, resulting in the production of antibodies that neutralized both the ancestral virus and BA.1 variant (12). Another study demonstrated that SARS-CoV-2 IgG increased two-fold in individuals who had received triple doses of mRNA vaccine and had no previous infection, at 2-5 weeks post Omicron infection (13). Additionally, the latest research has shown that BNT162b2 full vaccination reduced the risk of omicron-associated hospitalization by two-thirds among children who aged 5-11 years (14). In the study, among the unvaccinated children who were infected with SARS-CoV-2, the positive rate of SARS-CoV-2 Nabs was only 50.2%, and the majority of children had low expression levels. The results indicated that children had a weak humoral immune response against SARS-CoV-2 following natural infection.

In the realm, recent studies have compared the relationship between inactivated SARS-CoV-2 vaccines administered prior to or post infection and Nab levels in COVID-19 convalescent individuals. A study based on population over the age of 12 showed that the fully vaccinated recipients had higher levels of SARS-CoV-2 Nabs than the partially vaccinated and unvaccinated individuals, as well as a similar tendency was observed in positive rates (15).Another adult study revealed recipients of the CoronaVac vaccine exhibited significantly higher geometric mean Nabs titers and seropositivity rates in response to the Omicron variant compared to the unvaccinated in COVID-19 recovered patients (16). Recent research compared the effects of two doses of inactivated SARS-CoV-2 vaccine (CoronaVac) on previously naturally infected individuals and uninfected recipients. The results showed that anti-RBD antibody titers were significantly higher in those with a previous natural infection than in uninfected populations with two doses vaccine (17). The above results indicated that primary immunization prior to infection or complementary vaccination with two doses of inactivated SARS-CoV-2 vaccine during recovery from COVID-19 could effectively evoke immune memory effects and activate the humoral immune response to produce elevated antibodies.

In the landscape of SARS-CoV-2 Nabs detection, the traditional virus neutralization test and pseudovirus-based platforms, though classical methods of the field, present inherent challenges. Both methods require use of either live virus that requires a biocontainment level 3 laboratory, or extensive tissue culture capabilities, necessitating skilled technicians and protracted timelines, spanning several days to obtain conclusive results (18, 19). In the quest for more efficient and reliable alternatives, our study embraced a commercial Nabs assay, designed to gauge SARS-CoV-2 Nabs in the pediatric population following infection.

The innovative assay builds upon the RBD-ACE2 interaction, which was consistent with the principles of traditional viral neutralization testing and could detect specific Nabs in the serum of participants. Robustly validated by previous investigations, surrogate NAb levels have demonstrated a consistent correlation with live viral Nab titers, particularly against homologous ancestral viruses, attesting to their accuracy and relevance (20). A study focusing on analysis of serological biomarker showed that Nab of convalescent serum strongly correlated with Spike and RBD- specific antibody levels (21). Furthermore, a study has shown the correlation between the surrogate NAb with the ancestral virus and the Omicron variant was 0.908 and 0.797 respectively (15). Since there have been no previous outbreaks of SARS-CoV-2 in children in Beijing, the level of Nabs detected in the study may reflect the ability of Omicron variant infection to activate the humoral immune response in both vaccinated and unvaccinated children.

In navigating the scientific landscape, we acknowledge several limitations in the study. Firstly, it is vital to recognize that the Nabs detection reagent utilized in this investigation is a commercial kit, designed to target the RBD epitope of the SARS-CoV-2 S protein from the original strain. However, the dynamic nature of the virus has brought forth the emergence of the Omicron variant, featuring distinct genetic characteristics and mutations. Consequently, the commercial kit’s reliance on the original strain’s epitope may limit its ability to fully capture the specific neutralizing antibodies directed against the Omicron variant (22). Secondly, we confront the practicality of the lower limit for SARS-CoV-2 Nabs detection, set at 120.00 AU/ml. The constraints imposed by this threshold may lead to the underestimation of Nab levels in vaccinated children who subsequently encounter SARS-CoV-2 infection. Meanwhile, most children enrolled in the study experienced mild disease processes from SARS-CoV-2 infection, so we didn’t further analyze the correlation between the expression level of COVID-19 Nabs and the severity of the disease.

## 6 Conclusion

Within the realm of 3-11 years pediatric populations, children who had received two doses of the Corona Vac or BBIBP-CorV inactivated SARS-CoV-2 vaccine prior to infection exhibited a strikingly robust humoral immune response, characterized by the generation of an abundance of neutralizing antibodies. Even more remarkable was the sustained elevation of Nab levels for a notable period of 3 months post-infection regardless of age and gender. In contrast, children who remained unvaccinated before encountering the SARS-CoV-2 infection manifested a substantially weaker humoral immune response, with most individuals displaying inadequacy in generating Nabs. The SARS-CoV-2 infection acting as a potent enhancer, propelling the humoral immune response to new heights, bears profound significance for vaccine efficacy and immunization strategies in pediatric populations. Consequently, unvaccinated children who exhibit feeble humoral immunity following primary natural infection would largely benefit from timely intervention through vaccination, thereby establishing robust immunization protection against the ever-present risk of reinfection. However, the SARS-CoV-2 Omicron variant, adorned with pivotal mutations in the spike protein, ushers in current challenges, notably heightening the risk of reinfection and vaccine breakthrough events (23, 24). In light of these emerging uncertainties, the urgent call for long-term monitoring of reinfection cases and Nab levels in pediatric cohorts becomes evident. Such monitoring serves as a cornerstone for the formulation of evidence-driven vaccination strategies.

## Data availability statement

The original contributions presented in the study are included in the article/supplementary material. Further inquiries can be directed to the corresponding author.

## Ethics statement

The studies involving humans were approved by Ethics Committee of Capital Institute of Pediatrics. The studies were conducted in accordance with the local legislation and institutional requirements. Written informed consent for participation in this study was provided by the participants’ legal guardians/next of kin. Written informed consent was obtained from the minors’ legal guardian/next of kin for the publication of any potentially identifiable images or data included in this article.

## Author contributions

JL: Conceptualization, Data curation, Methodology, Project administration, Validation, Writing – original draft, Writing – review & editing. JJL: Data curation, Investigation, Writing – original draft. SD: Data curation, Writing – original draft. LD:Investigation, Writing – original draft. LW: Investigation, Writing – original draft. LC: Investigation, Writing – original draft. XC: Investigation, Writing – original draft. YW: Data curation, Writing – original draft. MG: Methodology, Writing – original draft. WL: Data curation, Writing – original draft. QS: Data curation, Writing – original draft. WX: Data curation, Writing – original draft. LM: Conceptualization, Validation, Writing – original draft, Writing – review & editing.
